# LncRNA NR-104098 Inhibits AML Proliferation and Induces Differentiation Through Repressing EZH2 Transcription by Interacting With E2F1

**DOI:** 10.3389/fcell.2020.00142

**Published:** 2020-03-26

**Authors:** Yubin Feng, Shuang Hu, Lanlan Li, Shengpeng Zhang, Jikang Liu, Xiaoling Xu, Meiju Zhang, Tianxi Du, Yan Du, Xiaoqing Peng, Feihu Chen

**Affiliations:** ^1^The Key Laboratory of Major Autoimmune Diseases of Anhui Province, Anhui Institute of Innovative Drugs, School of Pharmacy, Anhui Medical University, Hefei, China; ^2^The Key Laboratory of Anti-Inflammatory and Immune Medicines, Ministry of Education, Hefei, China; ^3^School of Pharmacy, Wannan Medical College, Wuhu, China

**Keywords:** long non-coding RNAs, acute myeloid leukemia (AML), E2F1, EZH2, NR-104098, proliferation, differentiation

## Abstract

Abundant evidence has illustrated that long non-coding RNA (lncRNA) plays a vital role in the regulation of tumor development and progression. Most lncRNAs have been proven to have biological and clinical significance in acute myeloid leukemia (AML), but further investigation remains necessary. In this study, we investigated lncRNA NR-104098 in AML and its specific mechanism. The microarray analysis was performed on NB4 cells. Based on the related analysis results, we identified that lncRNA NR-104098 is a suppressor gene that is significantly upregulated in AML cells. LncRNA NR-104098 could inhibit proliferation and induce differentiation in AML cells *in vitro* and also play main role in the mouse xenografts. Mechanically, it was confirmed that lncRNA NR-104098 may effectively inhibit EZH2 transcription by directly binding to E2F1 and recruiting E2F1 to the EZH2 promoter. In addition, ATPR can significantly increase the expression of lncRNA NR-104098, whereas knocking down NR104098 can inhibit the inhibitory effect of ATPR on the proliferation and induction differentiation of AML cells. Taken together, these results lead to deeper insight into the mechanism of ATPR-induced AML differentiation and prevent proliferation by inhibiting EZH2 on the transcriptional level.

## Introduction

As a very malignant hematological malignancy, acute myeloid leukemia (AML) accounts for a large proportion of leukemia. AML occurs due to the malignant proliferation of hematopoietic stem and progenitor cells (Thomas and Majeti, 2017; Pulikkan et al., 2018). The pathological features of AML are anemia, bleeding, infection, and, in more severe cases, death; these are caused by abnormal hematopoietic formations in the bone marrow and peripheral blood, in turn caused by the uncontrolled malignant proliferation of primitive cells (Marcucci et al., 2011; Stavropoulou et al., 2016). Generally, the high recurrence and mortality of AML is due to its complex pathogenesis (Sun et al., 2018; Zhang et al., 2018). In recent years, humans have made great progress in the diagnosis and treatment of AML, but the treatment effect is very unsatisfactory. Therefore, it is very urgent to further research the pathogenesis and potential therapeutic targets of AML. As the M3 typing of AML, the pathogenesis of acute promyelocytic leukemia (APL) is due to the translocation of chromosomes 15 and 17 to form the PML-RARA protein, which then blocks cell differentiation. Due to its high bleeding tendency and mortality, APL was once considered the most malignant type of AML (Friend et al., 1971; Breitman et al., 1980). Currently, retinoic acid (RA) and arsenic trioxide (ATO) are two classic drugs used for the treatment of APL. Over the years, all-trans retinoic acid (ATRA) resistance, relapse, differentiation syndrome, and adverse reactions have been challenges for APL treatment (Shen et al., 2004; Zuo et al., 2004; Meyer et al., 2008; Patatanian and Thompson, 2008). Simultaneously, ATRA seems to be a poor treatment option for non-APL. Thus, it is a matter of great urgency that researchers develop effective drugs to treat APL and non-APL. A new all-trans retinoic acid derivative, ATPR, was designed and synthesized by the School of Pharmacy, Anhui Medical University. Compared with ATRA, ATPR shows better solubility (Fan et al., 2014). In addition, ATPR is equally effective for APL (using NB4 cells) and non-APL (using THP-1 cells) (Feng et al., 2019a).

LncRNA is a non-coding RNA longer than 200 bp that does not have the ability to encode proteins (Chen et al., 2018). LncRNA can be classified into different types, such as sense, antisense, intronic, intergenic, and bidirectional (Ma et al., 2012). LncRNA regulates protein activity and subcellular localization of proteins by forming RNA protein complexes, thereby exerting physiological processes, such as gene expression, cell cycle, proliferation, and differentiation (Fatica and Bozzoni, 2014). LncRNA plays a key role in many types of diseases, including cancer, cardiovascular disease, autoimmune diseases, and nervous system diseases (Yang et al., 2014). Recently, more and more studies suggest that lncRNA may be closely related to leukemia (Gourvest et al., 2019; Wu et al., 2019). However, the role of lncRNA in the proliferation and differentiation of leukemia cells induced by ATPR is unclear.

To solve this problem, we used microarray analysis to investigate the expression of lncRNA in NB4 cells before and after ATPR induction. After ATPR-induced NB4 cells, 8662 lncRNAs and 9093 mRNAs with significant differential expression were screened. To explore the potential biological functions of these differentially expressed lncRNAs, we used Gene Ontology (GO) enrichment and Genes and Genomes (KEGG) for analysis. We established an lncRNA/mRNA co-expression network based on bioinformatic predictions and microarray results, and we then examined key transcription factors (TFs) associated with differentially expressed lncRNA. In addition, our results demonstrated the importance of a new lncRNA NR-104098 in the regulation of AML proliferation and differentiation.

In this study, we demonstrated that the lncRNA NR-104098 expression level was downregulated in AML cells. LncRNA NR-104098 inhibited the expression of EZH2 by interacting with the TF E2F1 to achieve proliferation inhibition and differentiation induction of AML. Moreover, lncRNA NR-104098 acted as a key factor in ATPR-inhibited AML proliferation and induced differentiation.

## Materials and Methods

### Reagents

ATPR (purity: 99.66%) was synthesized by the Pharmacology Laboratory of Anhui Medical University and dissolved in absolute ethanol (10^–2^ mol/L) and kept at −20°C.

### Cell Lines and Culture

We purchased NB4 and THP-1 cell lines from the Shanghai Gecko gene, and then cultured these in RPMI-1640 containing 10% fetal bovine serum (FBS) (Gibco, United States), penicillin (100 U/ml), and streptomycin (100 g/ml) −1640 medium.

We purchased lncRNA NR-104098 shRNA and negative scrambled shRNA from Hanbio (Shanghai, China). First, NB4 and THP-1 cells were seeded in 24-well plates before being transfected. Then, 30 μl shRNA was added to each well, allowed to stand at room temperature for 15 min, and placed in a cell culture incubator; the medium was changed after 24 h. pEGFP-C3-NR104098 were also purchased from Hanbio (Shanghai, China).

### Reverse Transcription Quantitative Polymerase Chain Reaction (RT-qPCR)

RNA samples were extracted from cells using chloroform and a Trizol reagent (Invitrogen, Carlsbad, CA, United States). SuperScript II (Vazyme, Nanjing, China) was used for the synthesis of Complementary DNA (cDNA). Amplification reactions were performed using a reaction system containing SYBR Green PCR Master Mix (Vazyme), 1 μL of cDNA, and amplification primers. β-Actin served as an internal control.

Several primers were used:

lncRNA NR-104098 forward, 5′-CCTGTATTTCTGCACCCG -3′;
lncRNA NR-104098 reverse, 5′-GCATGTTCTCACTCACGC-3′;
EZH2 forward, 5′-CCCTGACCTCTGTCTTACTTGTGGA-3′;
EZH2 reverse, 5′-ACGTCAGATGGTGCCAGCAATA-3′;
E2F1 forward, 5′-CGGCGATGTTACGACATTA-3′;
E2F1 reverse, 5′-CTTGTGGTAGTCTAGTTCTTGC-3′;
β-actin forward, 5′-CGCCGCCAGCTCACCATG-3′;
β-actin reverse, 5′-CACGATGGAGGGGAAGACGG-3′;

### Cell Viability Assay

Cell Counting Kit 8 (CCK8) (Bestbio, Shanghai, China) was used to measure cell proliferation capacity. The treated cells were seeded at 5000 cells per well in a 96-well plate, followed by overnight culture. Subsequently, the absorbance at 450 nm per well was detected using a microplate reader (Thermo Fisher Scientific). The number of viable cells was tested every 24 h for 3 days.

### Cell Cycle Analysis

The treated cells were collected, and the culture medium was removed by centrifugation and then fixed with pre-chilled 70% ethanol overnight. RNaseA was added to each tube and incubated in a 37°C water bath for 30 min; 400 ul propidium iodide (PI) was then added. CytoFLEX (Becton Dickinson, United States) was used to detect the cell cycle, and cycle distribution analysis was then performed with ModFIT software.

### Differentiation Marker Analysis

The treated cells were collected, the culture medium was removed by centrifugation, the cells were washed twice with PBS, and the cells were then incubated with CD11b (CD11b-PE/CY5) or CD14 (FITC) in the dark for 30 min on a shaker. CD11b and CD14 expression levels were measured using CytoFLEX (Becton Dickinson, United States).

### Western-Blot

The experimental method has been described previously (Feng et al., 2019b). Briefly, an equal amount of total protein was separated by SDS-PAGE gel and then subjected to other immunoblot analysis by antibody mouse anti-human β-actin (Cat No. bsm-33036M, Bioss, Beijing, China), rabbit anti-human E2F1 (Cat No. ab218527, Abcam, Danvers, MA, United States), rabbit anti-human CDK4 (Cat No. ab108357, Abcam, Danvers, MA, United States), rabbit anti-human Cyclin A2 (Cat No. ab181591, Abcam, Danvers, MA, United States), rabbit anti-human Cyclin D3 (Cat No. ab183338, Abcam, Danvers, MA, United States), rabbit anti-human P-Rb (Cat No. ab184796, Abcam, Danvers, MA, United States), rabbit anti-human CD11b (Cat No. ab133357, Abcam, Danvers, MA, United States), rabbit anti-human CD14 (Cat No. ab133335, Abcam, Danvers, MA, United States), and rabbit anti-human EZH2 (Cat No. ab186006, Abcam, Danvers, MA, United States). Immunoreactive bands were then visualized, and the optical densities were measured.

### Luciferase Reporter Assay

We commissioned Hanbio to prepare the human EZH2 luciferase reporter plasmid. We tested it according to the dual luciferase reporter gene specification (Promega, Shanghai, China). The cells were transfected, and then the Firefly and Renilla luciferase activities were measured. The activity of Firefly luciferase was normalized by Renilla luciferase.

### RNA Immunoprecipitation (RIP)

Cells were harvested, and nuclear proteins were extracted and then resuspended in RIP buffer. Immediately afterward, the resuspended RIP buffer was divided into three groups (for input as well as co-precipitation of IgG and E2F1 antibodies). Then, the supernatant was collected by centrifugation, and IgG (Abcam) or human anti-E2F1 antibody (Abcam) was added to each and then incubated at 4°C for 2 h. Subsequently, protein A beads were added and incubated for 1 h at 4°C. After centrifugation, the cells were washed three times with RIP buffer, then once with PBS, and the beads were resuspended in Trizol. Finally, quantitative detection was performed by reverse transcription quantitative polymerase chain reaction (RT-qPCR).

### RNA Pull-Down Assay

Biotin-labeled RNA pull-down was performed as described previously (Xiang et al., 2014). Briefly, nuclear proteins were extracted using a nuclear-plasma-isolation extraction kit and then incubated with biotin-labeled NR-104098 truncated probes and streptavidin agarose beads (Invitrogen). Finally, the retrieved protein was detected by Western-blot.

### Fluorescence *in situ* Hybridization (FISH)

In order to detect the lncRNA location, we performed fluorescence *in situ* hybridization (FISH) analysis, and cultured NB4 and THP-1 cells in NG medium containing lncRNA NR-104098 (genepharma, Shanghai, China) fluorescent probe as described previously (Li et al., 2018). 18S rRNA was used as a control probe for cytoplasmic control. Briefly, cells were fixed in 4% paraformaldehyde (Sigma), hybridized with a probe of lncRNA NR-104098 overnight, and then stained with DAPI. Fluorescence imaging was performed using a laser scanning confocal microscope.

### Chromatin Immunoprecipitation (ChIP)

We performed ChIP analysis according to the manufacturer of the EZ-ChIP kit (Upstate Biotechnology, Lake Placid, NY, United States) and then used this for the RT-qPCR assay.

### Tumor Xenografts

Six-week-old male NCG nude mice were purchased from the Nanjing Model Animal Institute. The mice were allowed to adapt in the SPF environment of Anhui Medical University (Hefei, China) for 1 week. The mice were randomly divided into two groups, and NB4 cells (1 × 10^6^) that were stably transfected with empty vector or pEGFP-C3-NR-104098 were injected subcutaneously. Eight weeks later, the mice were sacrificed, and the tumors were removed for weighing.

### Statistical Analysis

The data were expressed as the mean ± SD. Comparisons between multiple groups were made by one-way analysis of variance (ANOVA) followed by Duncan’s test. Differences were considered significant if the *p*-value was less than 0.05. The results shown were representative of at least three independent experiments.

### Microarray Analysis

Microarray analysis was performed by OE Biotech (Shanghai, China), and the specific method was described before (Chang et al., 2020). We performed three independent experiments and only considered significantly and repeated changes in lncRNA and mRNA as positive results.

## Results

### Differentially Expressed LncRNAs and mRNAs During ATPR-Inhibited NB4 Proliferation and Induced Differentiation

**FIGURE 1:**
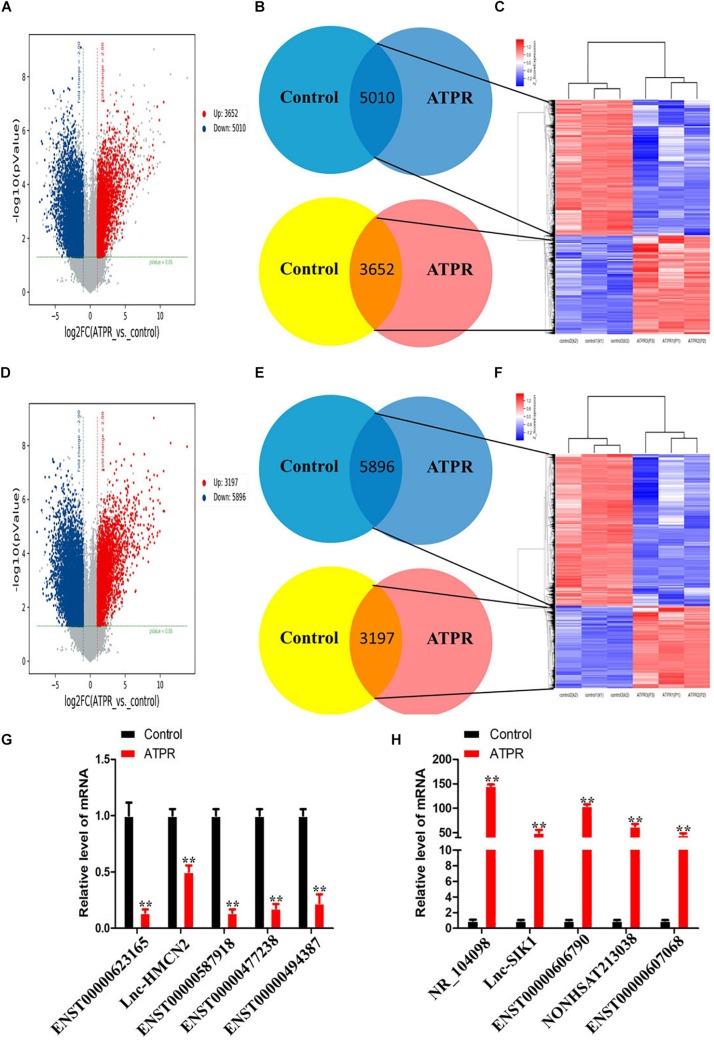
Differentially expressed lncRNAs and mRNAs during ATPR-inhibited NB4 proliferation and induced differentiation. **(A)** Volcano plots of differentially expressed lncRNAs relative to the control group. **(B)** LncRNAs that are up- and downregulated throughout ATPR-regulated NB4 proliferation and differentiation. **(C)** Heat map of lncRNAs up- and downregulated throughout ATPR-regulated NB4 proliferation and differentiation. **(D)** Volcano plots of mRNAs among those differentially expressed relative to the control group. **(E)** mRNAs that are up- and downregulated throughout ATPR-regulated NB4 proliferation and differentiation. **(F)** Heat map of mRNAs up- and downregulated throughout ATPR-regulated NB4 proliferation and differentiation. **(F)** Heat map of mRNAs up and downregulated throughout ATPR regulated NB4 proliferation and differentiation. **(G,H)** Validation of microarray data using q-PCR. Measurements for **(G)** five selected down-lncRNAs and **(H)** five selected up-lncRNAs.

We used microarray analysis to investigate whether induction of ATPR could affect lncRNA expression in NB4 cells. The intensity distribution was first normalized relative to the control, and volcano plots were then used to analyze differentially expressed lncRNAs and mRNAs (Figures 1A,D). The results showed that we identified 9,093 and 8,665 differentially expressed lncRNAs and mRNAs based on fold change ≥ 2.0 and *p*-value < 0.05 (*t*-test). According to the results of Figures 1B,C, compared with the control group, there were 3,652 upregulated lncRNAs and 5,010 downregulated lncRNAs in the ATPR group. Compared with the control group, 3,197 of the differentially expressed mRNAs were upregulated and 5,896 were downregulated (Figures 1E,F). qPCR was used to verify the expression of the top five often upregulated and downregulated lncRNAs, and the values of controls samples was fixed to 1 (Figures 1G,H).

### GO and KEGG Pathway Analyses of the Biological Functions of Genes Co-expressed With Differentially Expressed LncRNA

**FIGURE 2:**
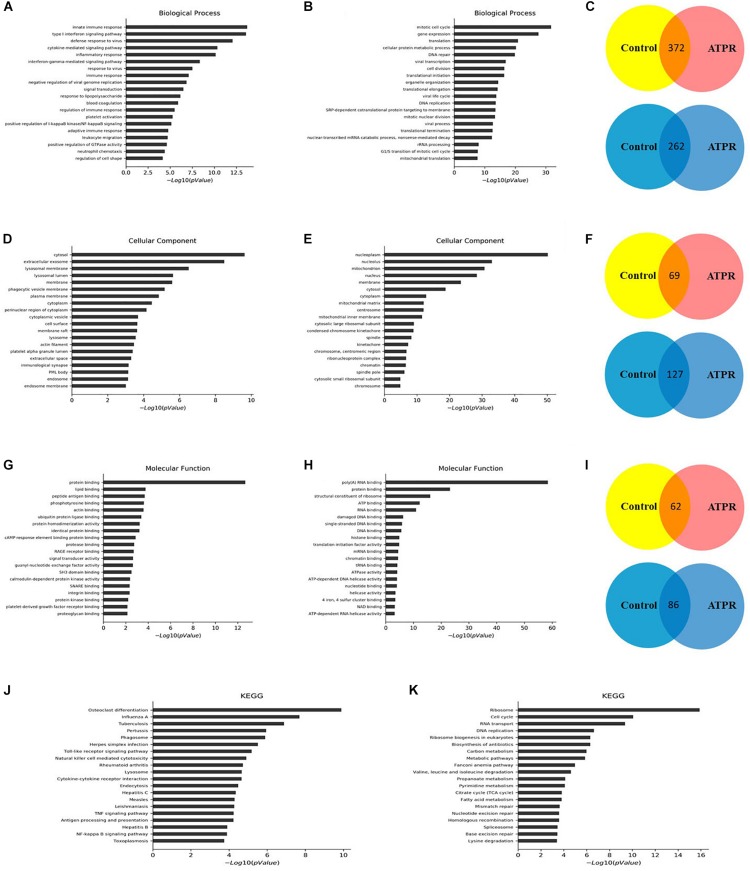
GO and KEGG pathway analyses of the biological functions of genes co-expressed with differentially expressed lncRNAs. Top 20 upregulated **(A)** and downregulated **(B)** BP terms during ATPR regulated NB4 proliferation and differentiation ranked by enrichment score. **(C)** BP terms up- or downregulated throughout ATPR-regulated NB4 proliferation and differentiation. Top 20 up- **(D)** and downregulated **(E)** CC terms during ATPR-regulated NB4 proliferation and differentiation ranked by enrichment score. **(F)** CC terms up- or downregulated throughout ATPR-regulated NB4 proliferation and differentiation. Top 20 up- **(G)** and downregulated **(H)** MF terms during ATPR-regulated NB4 proliferation and differentiation ranked by enrichment score. **(I)** MF terms up- or downregulated throughout ATPR-regulated NB4 proliferation and differentiation. Top 20 upregulated **(J)** and downregulated **(K)** pathways.

We used GO analysis to study the function of differentially expressed lncRNA during ATPR-induced NB4 differentiation. The results showed that, compared with the control group, there were 372 biological process (BP) terms, 69 CC terms, and 62 MF terms that were upregulated (*p* < 0.05) (Figures 2C,F,I), and 262 BP terms, 127 cellular component (CC) terms, and 86 molecular function (MF) terms that were downregulated (*p* < 0.05) (Figures 2C,F,I). Next, the top 20 important GO terms were classified and ranked according to the enrichment scores (Figures 2A,B,D,E,G,H). The most enriched and significantly upregulated BP terms were related to the innate immune response, type I interferon signaling pathway, defense response to virus, cytokine-mediated signaling pathway, inflammatory response, and interferon-gamma-mediated signaling pathway (Figure 2A); the most enriched upregulated CC terms were associated with cytosol, extracellular exosome, lysosomal membrane, lysosomal lumen, and membrane (Figure 2D); and the most enriched upregulated MF terms were related to protein binding, lipid binding, peptide antigen binding, phosphotyrosine binding, and actin binding (Figure 2G). In contrast, the most highly enriched downregulated BP terms were mitotic cell cycle and gene expression (Figure 2B); the most enriched CC terms included nucleoplasm and nucleolus (Figure 2E), and the most enriched MF terms involved poly(A) RNA binding, protein binding, and structural constituents of the ribosome (Figure 2H).

To identify the key factors in the process of AML-induced differentiation, we used KEGG for analysis. Significantly altered pathways were selected based on *p*-value < 0.05, and these pathways were ranked based on the number of genes. Analysis results showed that 40 and 43 pathways are associated with upregulated and downregulated genes, respectively (Figures 2J,K). The cell cycle, ribosome, osteoclast differentiation, and influenza A pathways may play important roles in this process.

### Construction of the LncRNA-mRNA Co-expression Network

**FIGURE 3:**
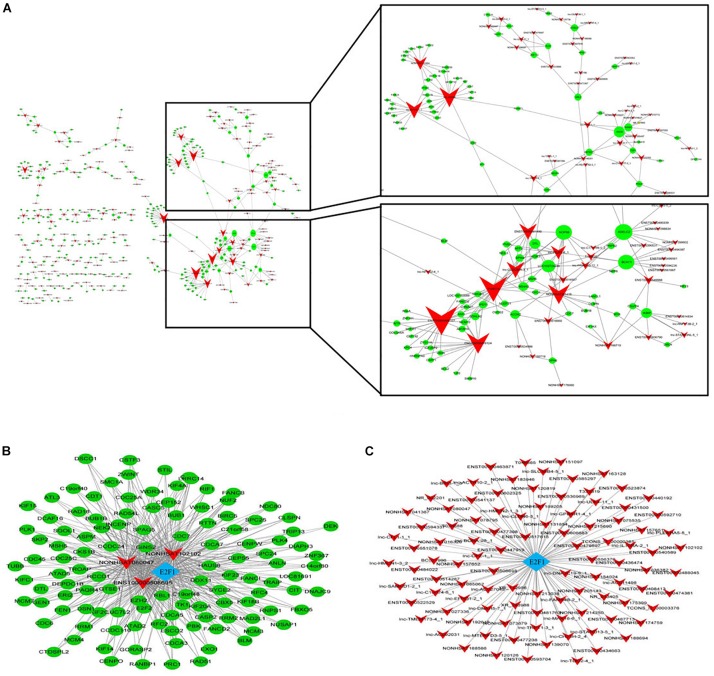
Construction of the lncRNA-mRNA co-expression network. Circle and square nodes represent mRNAs and lncRNA, respectively. The sizes of circle nodes are proportional to the number of interacting lncRNA. **(A)** Core network map constructed based on the 500 most significantly correlated lncRNA/mRNA pairs. **(B,C)** Correlation analysis between lncRNA and TFs. Arrow, rhombus, and circle nodes represent lncRNA, TFs, and target genes, respectively. **(B)** Network map of the top 200 lncRNA/TF pairs. The size of rhombus nodes is proportional to the number of interacting lncRNA. **(C)** Network map of the top 200 lncRNA/TF/target gene interactions. The sizes of rhombus and circle nodes are proportional to the number of interacting TFs and lncRNA, respectively.

In order to determine the interaction relationship between lncRNA and mRNA in the difference table, we screened the most significantly 500 lncRNA and mRNA pairs to construct a coding/non-coding gene co-expression network (Figure 3A). When examining lncRNA function, “trans” regulatory mechanisms that affect chromatin, transcription, or other processes must be considered. As a step in this direction, co-expressed lncRNA-mRNA pairs were examined to identify mRNAs that encode known TFs. A complete list of TF-lncRNA pairs is shown in Supplementary Table 1. Because the number of TF-lncRNA pairs is so large (>3329), it is not practical to generate a network directly from this data. To simplify the network, we selected the top 200 pairs to generate a core network map (Figure 3B). Most of the predicted trans-regulatory lncRNA participate in pathways regulated by only 1 TF, E2F1. The interaction network shows that all lncRNAs that play a role in ATPR revolve around a TF E2F1, which may play an important role in the treatment of AML by ATPR. The top 200 co-expressed gene pairs were also used to construct a lncRNA-TF-mRNA network. The network structure, shown in Figure 3C, suggested that the TFs E2F1 along with three lncRNA are transcriptional regulators that play important roles in various signal pathways.

### ATPR Induced the Expression of LncRNA NR-104098

**FIGURE 4:**
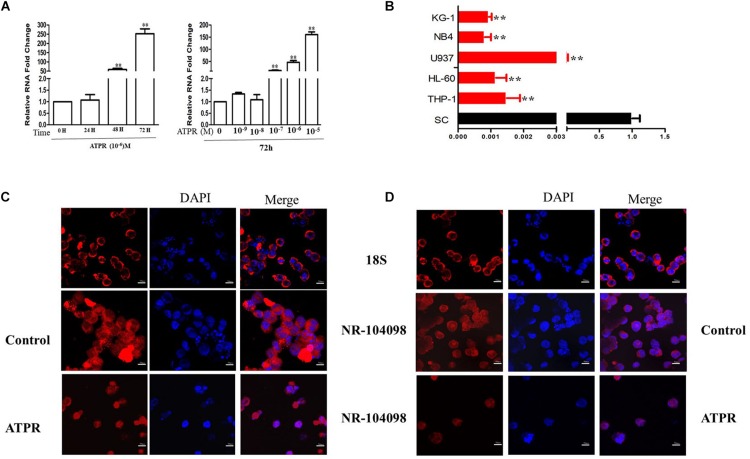
ATPR Induced the Expression of lncRNA NR-104098. **(A)** NB4 cells were treated with ATPR (10^–6^ M) at different time points (0, 24, 48, and 72 h). NB4 cells were treated with an ATPR concentration gradient (0–10^–5^ M) for 72 h. Then, the mRNA expression of lncRNA NR-104098 were assessed by RT-PCR. **(B)** RT-PCR analysis of lncRNA NR-104098 expression in AML cell lines and SC cells. **(C,D)** lncRNA NR-104098 distribution detected by FISH. Values were presented as mean ± SD of three independent experiments. *p < 0.05, **p < 0.01 versus SC cells group.

The NCBI database showed that lncRNA NR-104098 belongs to a new lncRNA. LncRNA position prediction website results showed that lncRNA NR-104098 is located in the cytoplasm^1^. In addition, qPCR results revealed that ATPR induced expression of lncRNA NR-104098 in a time- and concentration-dependent manner (Figure 4A). Besides, the lncRNA NR-104098 expression was significantly reduced in a number of leukemia cell lines compared with in the normal human monocytes (Figure 4B). We further confirmed the expression of lncRNA NR-104098 using the FISH method, labeled the nuclei with DAPI, and labeled lncRNA NR-104098 with CY3 and 18S rRNA (cytoplasmic positive). The results showed that lncRNA NR-104098 was localized in the cytoplasm of NB4 and THP-1 cells. LncRNA NR-104098 transferred to the nucleus after ATPR treatment (Figures 4C,D). These data suggest that lncRNA NR-104098 may be a ATPR-related factor.

### LncRNA NR-104098 Overexpression Suppressed Proliferation and Induced Differentiation of AML Cells *in vitro*

**FIGURE 5:**
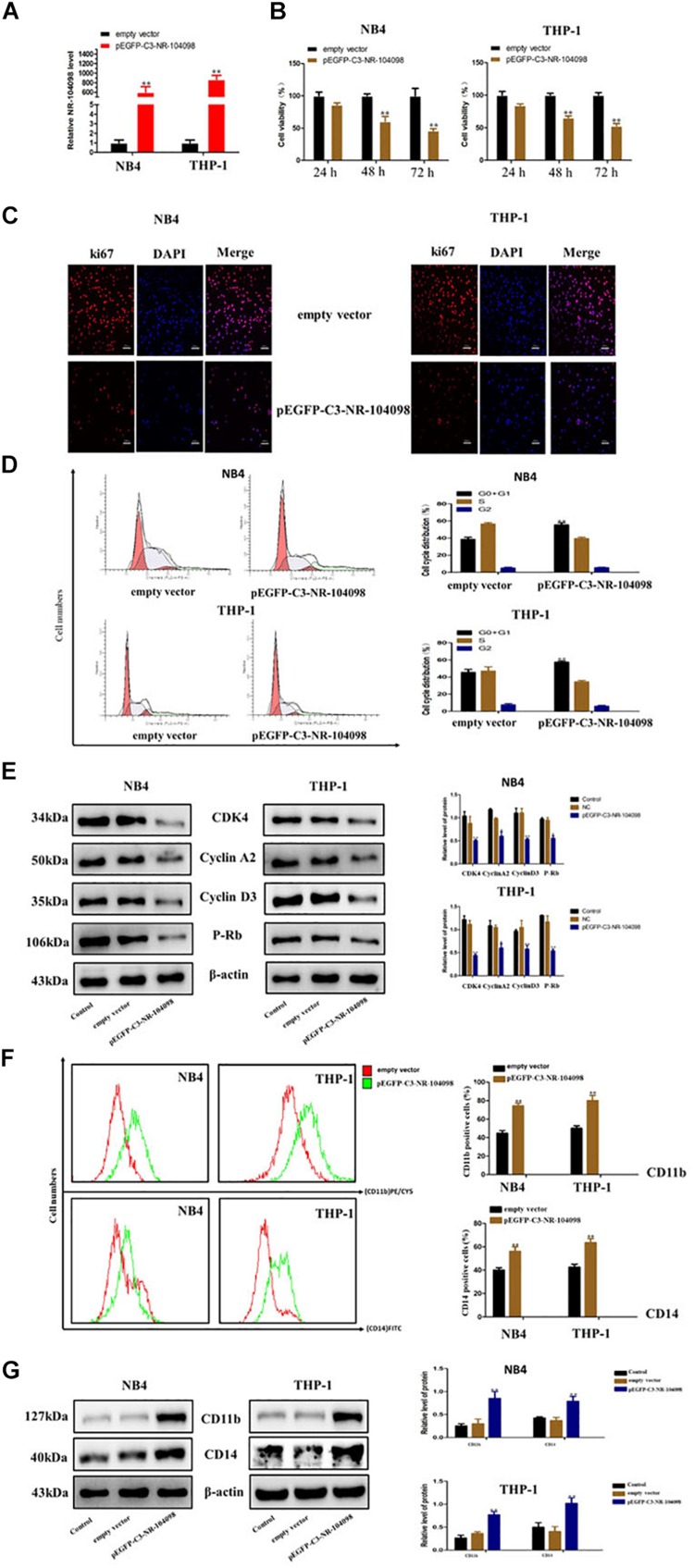
LncRNA NR-104098 overexpression suppressed proliferation and induced differentiation of AML cells *in vitro*. **(A)** pEGFP-C3-NR-104098 or the empty vector was stably transfected into AML cells. 48 h later, overexpression efficient of lncRNA NR-104098 was evaluated by RT-qPCR. **(B)** pEGFP-C3-NR-104098 or the empty vector was transfected into AML cells for 24, 48, and 72 h respectively, and cell viabilities were assessed by CCK8 assay. **(C)** pEGFP-C3-NR-104098 or the empty vector was transfected into AML cells for 48 h, and ki67 protein expression was detected by immunofluorescence. **(D)** pEGFP-C3-NR-104098 or the empty vector was transfected into AML cells for 48 h, and the cell cycle were analyzed by flow cytometry. **(E)** Western blot analysis of cyclin D3, cyclin A2, P-rb, and CDK4 protein levels in AML cells after pEGFP-C3-NR-104098 or the empty vector was transfected into AML cells. **(F)** pEGFP-C3-NR-104098 or the empty vector was transfected into AML cells for 48 h, and cell differentiation were analyzed by flow cytometry. **(G)** Western blot analysis of CD11b and CD14 protein levels in AML cells after pEGFP-C3-NR-104098 or the empty vector was transfected into AML cells. β-Actin served as a loading control. Data are presented as the mean ± SD of three independent experiments. ^∗^*p* < 0.05, ^∗∗^*p* < 0.01, significant difference between pEGFP-C3-NR-104098 and the empty vector. These observations demonstrated that silencing lncRNA NR-104098 could inhibit the ATPR’s effects to a certain extent. Thus, lncRNA NR-104098 might be a crucial factor in the anti-cancer effect of ATPR in AML cells, which also suggested that lncRNA NR-104098 could be an attractive tumor suppressor molecule in future AML treatment.

Given that lncRNA NR-104098 was downregulated in AML cells in this study, we further explored the effects of lncRNA NR-104098 on AML cell biological activity. The mRNA expression levels of lncRNA NR-104098 were significantly increased in both NB4 and THP-1 cells after transfection with pEGFP-C3-NR104098 (Figure 5A). CCK8 results indicated that over-expressed lncRNA NR104098 can inhibit cell proliferation of NB4 and THP-1 cells in a time-dependent manner (Figure 5B). To evaluate the effect of lncRNA NR-104098 on proliferation in AML cell lines, the expression of the proliferation-associated protein ki67 was determined by immunofluorescence staining analysis (Figure 5C). On the other hand, flow cytometric assays were performed to study the effect of lncRNA NR-104098 on the cycle arrest of AML cell lines. Forced expression of lncRNA NR-104098 induced G0/G1 cycle arrest of NB4 and THP-1 cells (Figure 5D). Consistently, 72 h after transfection of pEGFP-C3-NR104098, Western-blot assays were performed and G0/G1 signature protein was found to be decreased in both AML cell lines (Figure 5E). We previously studied the effects of lncRNA NR-104098 on cell proliferation. It is a well-known fact that the pathogenesis of leukemia is blocked by differentiation. A previous study showed that CD11b and CD14 were relatively classic markers of differentiation in leukemia. We used a series of experimental methods to detect whether lncRNA NR-104098 could affect leukemia cell differentiation. The flow cytometry results showed that overexpression of lncRNA NR-104098 promoted CD11b (PE/CY5) and CD14 (FITC) expression of NB4 and THP-1 cells (Figure 5F). The results showed that overexpression of lncRNA NR-104098 promoted CD11b and CD14 protein expression in NB4 and THP-1 cells (Figure 5G). Taken together, these data suggested that overexpression lncRNA NR-104098 may inhibit AML proliferation and induce differentiation.

### Knockdown of LncRNA NR-104098 Inhibited ATPR-Suppressed Proliferation and Induced Differentiation of AML Cells *in vitro*

**FIGURE 6:**
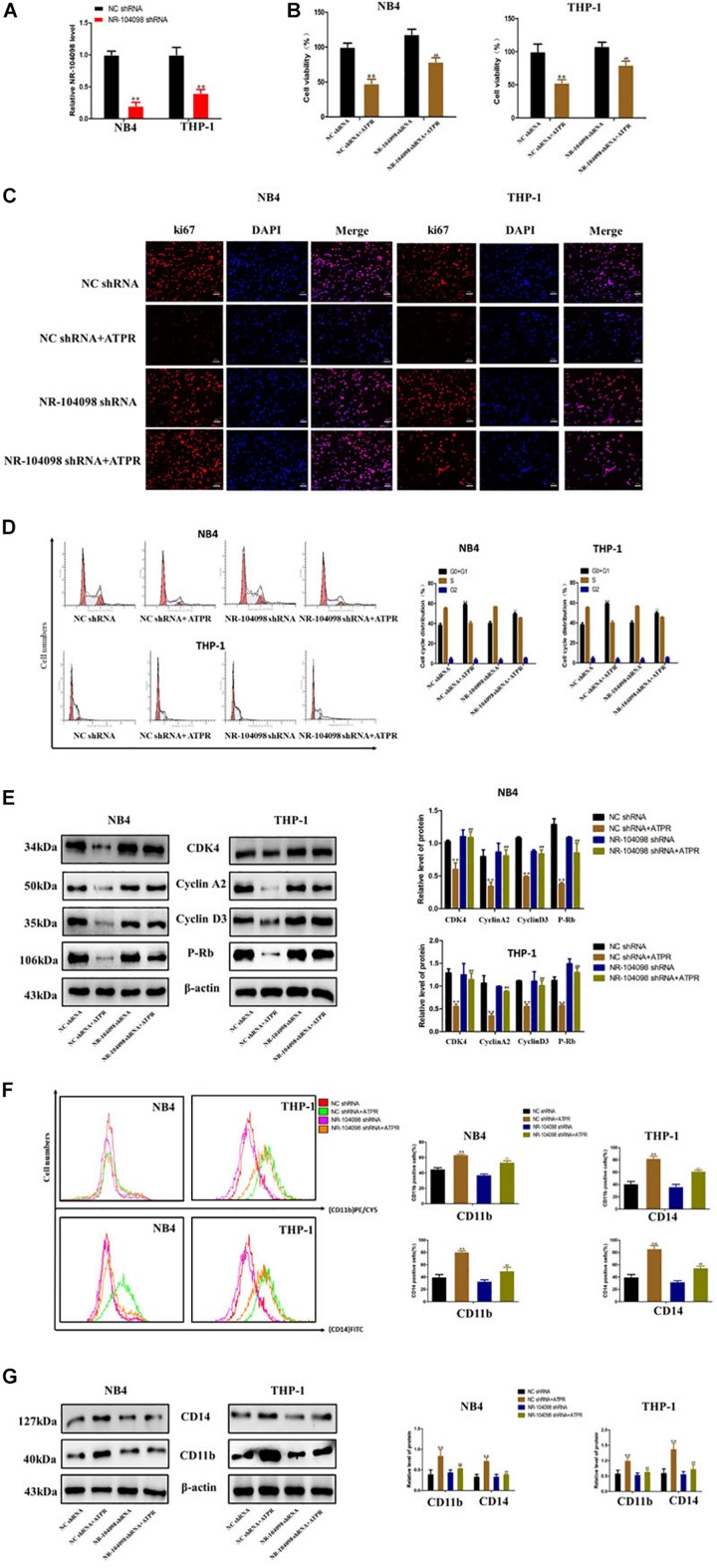
Knockdown of lncRNA NR-104098 inhibited ATPR-suppressed proliferation and induced differentiation of AML cells *in vitro*. **(A)** The expression levels of lncRNA NR-104098 in NB4 and THP-1 cells were detected by RT-qPCR assay, with transfection of lncRNA NR-104098 shRNA or the NC shRNA for 48 h. **(B)** AML cells were treated with ATPR or Solvent. 72 h later, cells were collected and cell viabilities were evaluated by CCK8 assay. **(C)** Meanwhile, ki67 protein expression detected by immunofluorescence. **(D)** Cell cycle were analyzed by flow cytometry analyzed. **(E)** Western blot analysis of cyclin D3, cyclin A2, P-rb, and CDK4 protein levels in AML cells after treated. **(F)** Cell differentiation were analyzed by flow cytometry. **(G)** Western blot analysis of CD11b and CD14 protein levels in AML cells after treated. β-Actin served as a loading control. Data are presented as the mean ± SD of three independent experiments. ^∗^*p* < 0.05, ^∗∗^*p* < 0.01, compared with the NC shRNA group. ^#^*p* < 0.05,^##^*p* < 0.01, compared with the ATPR group.

ATPR is a novel and promising anti-cancer compound for AML (Feng et al., 2019a, b). Here, we investigated the potential role of lncRNA NR-104098 in anti-cancer compound ATPR-suppressed proliferation and induced differentiation of AML cells. We explored whether lncRNA NR-104098 can affect ATPR-suppressed proliferation and induced differentiation of AML cells. NR104098-shRNA was transfected into AML cells and effectively suppressed NR104098 levels (Figure 6A). CCK8 assays also showed that knockdown of lncRNA NR-104098 ameliorated ATPR-inhibited cell proliferation (Figure 6B). Immunofluorescence staining analysis results showed that lncRNA NR-104098 can increase the ATPR-induced proliferation-related protein ki67 (Figure 6C). Flow cytometric assays also showed that knockdown of lncRNA NR-104098 inhibited ATPR-induced cell G0/G1 cycle arrest (Figure 6D). After NR104098-shRNA was transfected into AML cells, P-rb, cyclin D3, cyclin A2, and CDK4 were upregulated (Figure 6E) to analyze whether lncRNA NR-104098 is closely involved in ATPR-induced differentiation of AML cells. Flow cytometry results also showed that compared with the ATPR group, NR104098-shRNA can significantly downregulate the expression of CD11b (PE/CY5) and CD14 (FITC) (Figure 6F). Western blot results also showed that after silencing lncRNA NR-104098, the expression of CD11b and CD14 induced by ATPR was reduced in AML cells (Figure 6G).

### Upregulation of LncRNA NR-104098 Inhibited EZH2 Transcription in AML Cells

**FIGURE 7:**
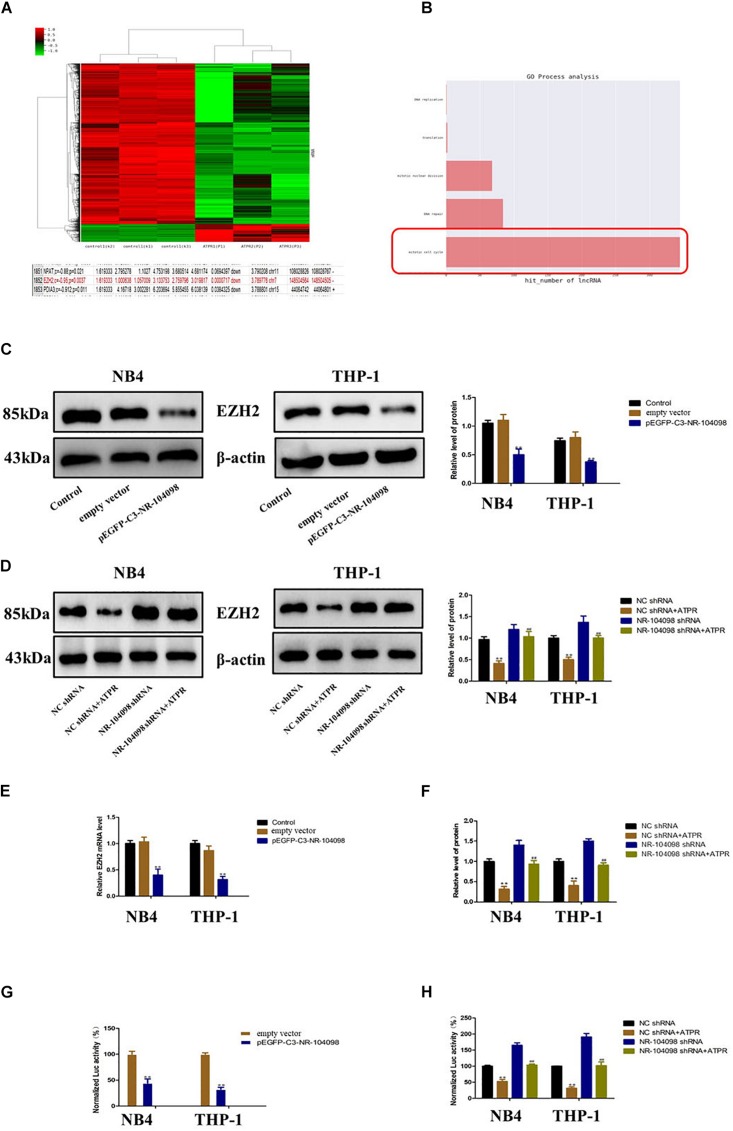
Upregulation of lncRNA NR-104098 inhibited EZH2 transcription in AML cells. **(A)** Microarray analyses results showed that lncRNA NR-104098 may regulate the oncogene EZH2. **(B)** GO process analysis results of lncRNA NR-104098 regulates cell cycle. **(C)** pEGFP-C3-NR-104098 or the empty vector was stably transfected into AML cells, and EZH2 protein levels were detected by Western-blot assay. **(D)** lncRNA NR-104098 shRNA or the NC shRNA was stably transfected into AML cells, the ATPR or Solvent was treated for 72 h, and the Western-blot assay indicated that lncRNA NR-104098 shRNA evidently inhibited ATPR-induced downregulation of EZH2 protein expression. **(E)** Either AML cells stably transfected with pEGFP-C3-NR-104098 or the empty vector were collected, and EZH2 mRNA levels were assessed by RT-qPCR assay. **(F)** Either AML cells stably transfected with lncRNA NR-104098 shRNA or NC shRNA were treated with ATPR or Solvent for 72 h, RT-qPCR showed that lncRNA NR-104098 shRNA remarkably reversed the inhibition of EZH2 mRNA expression by ATPR. **(G)** Either EZH2 transcriptional activities of AML cells stably transfected with pEGFP-C3-NR-104098 or the empty vector were assessed by luciferase reporter assay. **(H)** The cells were then treated with ATPR or Solvent. 72 h later, EZH2 transcriptional activities were analyzed, and the activity of firefly luciferase was normalized by luciferase reporter assay. β-Actin served as a loading control. Data are presented as the mean ± SD of three independent experiments. ^∗^*p* < 0.05, ^∗∗^*p* < 0.01, compared with the NC group. ^#^*p* < 0.05, ^##^*p* < 0.01, compared with the ATPR group.

Our microarray analyses results showed that lncRNA NR-104098 may regulate the oncogene EZH2 (Figure 7A). And further GO process analysis results also show that lncRNA NR-104098 regulates cell cycle (Figure 7B).

Therefore, we investigated whether lncRNA NR-104098 affects the expression of EZH2. Overexpression of lncRNA NR-104098 can significantly downregulate the EZH2 protein level (Figure 7C). As shown in Figure 7D, compared with the negative control, silencing lncRNA NR-104098 could reverse ATPR-induced downregulation of EZH2 protein. Overexpression of lncRNA NR-104098 can also inhibit EZH2 mRNA expression (Figure 7E), and knockdown of lncRNA NR-104098 restored ATPR-induced suppression of EZH2 mRNA expression in NB4 and THP-1 cells (Figure 7F). To further elucidate the specific mechanism of lncRNA NR-104098-regulating EZH2 expression, we used a human EZH2 luciferase reporter gene plasmid for the luciferase reporter gene analysis. Overexpression of lncRNA NR-104098 can reduce EZH2 promoter activity in NB4 and THP-1 cells (Figure 7G). Knocking down lncRNA NR-104098 can also reverse the downregulation of EZH2 mRNA transcription activity induced by ATPR (Figure 7H). These results proved that lncRNA NR-104098 negatively regulated EZH2 transcription in AML cells.

### LncRNA NR-104098 Downregulated Transcriptional Expression of EZH2 Through Recruitment of E2F1 in AML Cells

**FIGURE 8:**
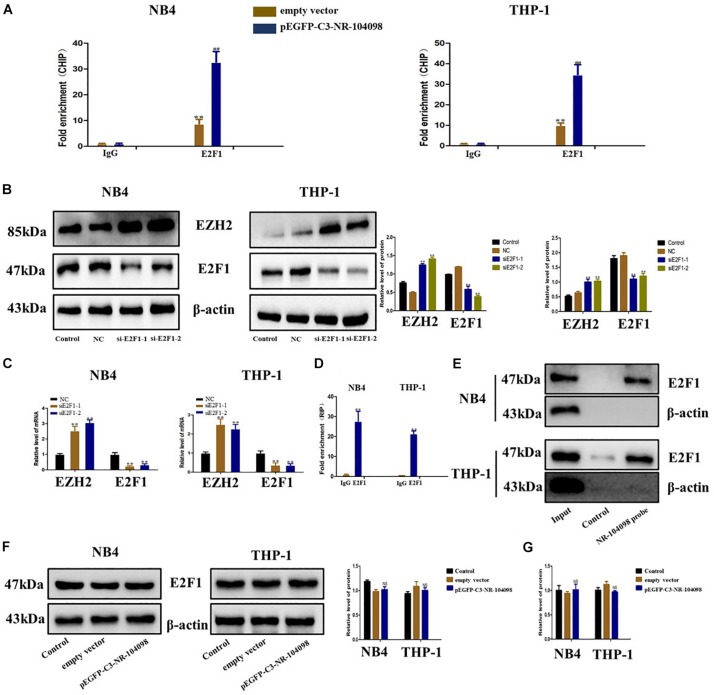
lncRNA NR-104098 downregulated transcriptional expression of EZH2 through recruitment of E2F1 in AML cells. **(A)** pEGFP-C3-NR-104098 or the empty vector was transfected into AML cells, and chrome immunoprecipitations were performed by using specific anti-E2F1 antibodies. **(B)** AML cells transfected with E2F1 siRNAs or the control siRNA for 72 h were collected, and EZH2 and E2F1 protein levels were detected by Western-blot assay. **(C)** E2F1 siRNAs (E2F1-1, 2) or the control siRNA were transfected into AML cells for 48 h, E2F1 and EZH2 mRNA levels were then assessed by RT-qPCR. **(D)** RNA immunoprecipitations were performed in AML cells, and the relative quantities of lncRNA NR-104098 were detected by RT-qPCR assay, normalized to the input groups. IgG and E2F1 represented for the groups coprecipitation with IgG protein and anti- E2F1 antibody respectively. **(E)** Total proteins were extracted from NB4 and THP-1 cells, and then lncRNA NR-104098 pull-down assay was performed. The E2F1 protein levels were evaluated by Western-blot. LncRNA NR-104098 probe represented the biotin-labeled lncRNA NR-104098 probe group and control stood for the oligo probe group. NB4 and THP-1 cells with pEGFP-C3-NR-104098 or the empty vector was harvested, and expression levels of E2F1 protein **(F)** and E2F1 mRNA **(G)** were detected. β-Actin served as a loading control. Data are presented as the mean ± SD of three independent experiments. ^∗^*p* < 0.05, ^∗∗^*p* < 0.01, compared with the NC group. NS no significant difference between the groups, ^#^*p* < 0.05, ^##^*p* < 0.01, significant difference between pEGFP-C3-NR-104098 and the empty vector.

It has been reported in the literature that the transcription of EZH2 is regulated by a variety of TFs (such as E2Fs). Our CHIP analysis results show that overexpression of lncRNA NR-104098 can significantly improve the binding capacity of E2F1 (Figure 8A). Our previous predictions suggested that E2F1 may be a key TF regulating AML. We used E2F1 siRNA to silence E2F1 expression. RT-qPCR and western blot showed that EZH2 expression was significantly upregulated (Figures 8B,C). Subsequently, we performed an RNA immunoprecipitation (RIP) analysis, and the results showed that E2F1 protein can bind to lncRNA NR-104098 (Figure 8D). The direct interaction between E2F1 and lncRNA NR-104098 was further proved by applying total protein (Figure 8E) to lncRNA NR-104098 pull-down assay. In addition, we found lncRNA NR-104098 did not impact the expression of E2F1 protein and E2F1 mRNA (Figures 8F,G). Taken together, we concluded that lncRNA NR-104098 inhibited EZH2 transcriptional activity by recruiting TF E2F1 to the EZH2 promoter.

### Upregulation of LncRNA NR-104098 Suppressed Proliferation and Induced Differentiation of AML Cells *in vivo*

**FIGURE 9:**
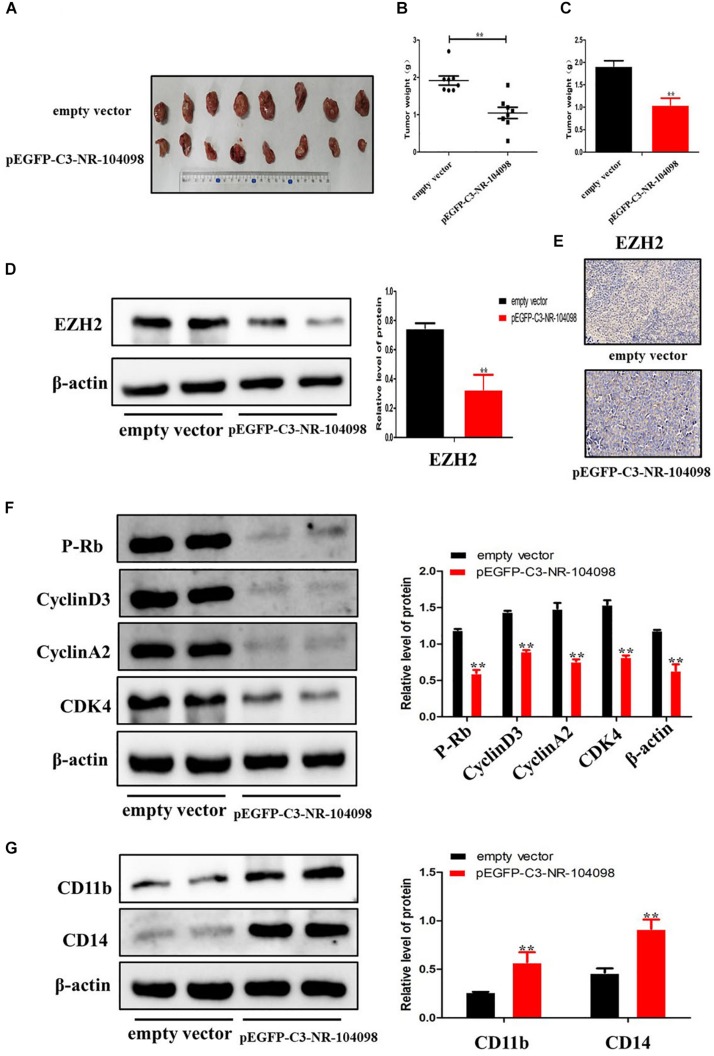
Upregulation of lncRNA NR-104098 suppressed proliferation and induced differentiation of AML cells *in vivo*. Four-week-old NCG mice were randomly divided into two groups, and NB4 cells (1 × 10^6^) with pEGFP-C3-NR-104098 or empty vector stable transfection were injected s.c. respectively. 8 weeks after that, the mice were sacrificed and the tumors were removed, photographed **(A)**, and weighed **(B,C)**. **(D)** Then, protein was extracted from the tumor tissues, and expressions of EZH2 protein was detected by Western blot. **(E)** Immunohistochemistry analysis of EZH2 was obtained from tumors. **(F)** Western blot analysis of cyclin D3, cyclin A2, P-rb, and CDK4 in tumor tissues of pEGFP-C3-NR-104098 or empty vector groups. **(G)** Western blot analysis of CD11b and CD14 in tumor tissues of pEGFP-C3-NR-104098 or empty vector groups. β-Actin served as a loading control. Data are presented as the mean ± SD of three independent experiments. ^∗^*p* < 0.05, ^∗∗^*p* < 0.01, significant difference between pEGFP-C3-NR-104098 and the empty vector.

In order to confirm the effect of lncRNA NR-104098 on tumor formation *in vivo*, nude mice received subcutaneous injections of pEGFP-C3-NR104098 treated or empty vector NB4 cells to establish the tumor model. After 8 weeks, tumors were completely stripped. Photographs and weight of the tumors indicated that lncRNA NR-104098 overexpression cells grew much more slowly than empty vector group cells (Figures 9A–C). Moreover, immunohistochemistry and Western-blot indicated that lncRNA NR-104098 downregulated the EZH2 protein (Figures 9D,E). Western blotting showed that lncRNA NR-104098 overexpression inhibited the expression of cyclin D3, cyclinA2, P-rb, and CDK4 and promoted the expression of CD11b and CD14 compared with the expression in control tumors (Figures 9F,G). Together, these data indicated that, as in the *in vitro* experiments, lncRNA NR-104098 may affect the proliferation and differentiation of AML tumors *in vivo*.

## Discussion

Although high-throughput gene sequencing technology has been widely used, and many new lncRNA have been identified, they are involved in gene expression regulation in normal physiological and disease states (Yan et al., 2012), and only a few lncRNAs have been shown to be involved in AML. ATPR, as a new drug researched by our research group, not only has a better therapeutic effect on leukemia but also better solubility. In this study, we treated NB4 with ATPR and analyzed the expression profile of lncRNA during ATPR-inhibited proliferation and induced differentiation. Our results showed that many more differentially expressed lncRNAs and mRNAs were identified compared to the control group after ATPR-induced AML differentiation. Interestingly, we observed that many lncRNAs and mRNAs were more highly differentially expressed in AML than before induction. As a new lncRNA, NR-104098, may play an important role in AML-inhibited proliferation and induced differentiation. Upregulation of lncRNA NR104098 inhibited proliferation and induced differentiation, whereas its downregulation inhibited ATPR-affected AML proliferation and differentiation.

To predict the function of differentially expressed lncRNA, we performed a GO enrichment analysis and KEGG analysis. GO enrichment results showed that the top GO terms were related to the differentiation function. For example, the most pronounced upregulation of BP, CC, MF, and KEGG terms was related to physiological processes, such as innate immune response, cytosol, protein binding, and osteoclast differentiation. In contrast, the most pronounced downregulation of BP, CC, MF, and KEGG terms were related to the mitotic cell cycle, nucleoplasm, poly(A) RNA binding, and Ribosome. The lncRNA-mRNA co-expression network results showed that essential signal molecules (such as MSH6, KDELC2, BCAT1, ANO6, and KANK1) may be related to a variety of lncRNA, including NONHSAT131854, NR033856, LNST00000432120, and ENST00000495327. This suggests that lncRNA may play a critical role in AML proliferation and differentiation by regulating the expression of specific target mRNAs. MSH6 mutation is associated with the development of medulloblastoma or AML (Scott et al., 2007). BCAT1 affects AML stem cell methylation by regulating αKG levels (Raffel et al., 2017). In our study, one candidate lncRNA NR-104098 was selected to investigate its function during AML proliferation and differentiation. Our results showed that lncRNA NR-104098 expression increases dramatically during ATPR induced.

Leukemia is caused by a block in differentiation. The treatment of this disease is mainly induced differentiation therapy (Miyazawa et al., 2001; Muto et al., 2001; Funato et al., 2002; Rizvi and Gores, 2013; Feng et al., 2019a, b). Differentiation is an important regulatory mechanism of AML. During the differentiation and maturation of AML, the antigenic markers on the surface of hematopoietic cells will be changed. Different stages of cell differentiation can be labeled with different antigens. CD11b, as a differentiation marker of mature granulocytes, is expressed in neutrophils, basic granulocytes, acid granulocytes, and monocytes (Mason et al., 2006). CD14 is also a marker of differentiation of mature granulocytes and mainly expressed in monocytes (Ziegler-Heitbrock and Ulevitch, 1993). Our results indicated that the upregulation of lncRNA NR-104098 can significantly promote the expression of CD11b and CD14, indicating that AML cells tended to differentiate into mature granulocytes and monocytes. The cell cycle is a series of events that lead to cell division and replication. Generally, DNA damage, nutrient depletion, and withdrawal of growth factors can all affect DNA replication, which in turn affects the cell cycle (Otto and Sicinski, 2017; Pietrzak et al., 2018). The G1 phase of cells is regulated by the interaction between cyclins and cyclin-dependent kinase (CDK), which in turn affects cell division (Hydbring et al., 2016; Swaffer et al., 2016), which are important for driving cells past checkpoints (Marchini et al., 1988). Importantly, obstacles to cell proliferation and differentiation are the two most important features of hematological malignancies. Targeting the cell cycle, proliferation and differentiation is a potential approach to treating leukemia. We found that overexpression of lncRNA NR104098 inhibits the proliferation and induces the differentiation of AML. At the same time, our results also show that knocking down lncRNA NR104098 show the opposite effects in AML cells. Next, it was showed that overexpression of lncRNA NR104098 induced cell cycle arrest in the G0/G1 phase. However, studies on the role of lncRNA NR104098 on apoptosis and drug resistance in AML have not been carried out, and this role remains to be investigated.

Among all the significantly upregulated lncRNAs, we chose the most upregulated lncRNA NR-104098 for further study. We found that lncRNA NR-104098 expression levels were significantly reduced in AML cell lines compared to normal cell line expression levels. Mechanically, the effects of lncRNA NR-104098 overexpression and silencing on differentiation and appreciation were identified in order to study their relevance and the possible underlying mechanisms of their action. Furthermore, our results indicated that upregulation of lncRNA NR-104098 inhibits the proliferation and induces the differentiation of AML cells both *in vivo* and *in vitro*. We also found that lncRNA NR-104098 inhibited EZH2 transcription by binding to the TF E2F1 and recruiting it to the EZH2 promoter, and silencing lncRNA NR-104098 can reverse the induction of AML by ATPR. These findings shed new light on a novel tumor-suppressing mechanism for lncRNA NR-104098 in AML. Although a large part of the function of lncRNA has yet to be developed, new research results showed that lncRNA can bind to TFs to activate or inhibit gene expression (Hung et al., 2011; Zhu et al., 2015). Previous study found that GAS5 can bind to the TF glucocorticoid receptor (GR) and titrate it away from its target gene promoters (Kino et al., 2010). Results of RNA-seq data showed that lncRNA can regulate the expression of cell cycle-related genes and regulate the disease process of breast cancer by combining with E2F TFs (Sun et al., 2015). E2F activity is regulated by pocket proteins, which can bind to E2F in a form lacking in active phosphate and inhibit the transcription of E2Fs target genes (Zhan et al., 2014). Recent research results also showed that lncRNA GAS5 can interact with the TF E2F1 and enhance E2F1 binding to the cell cycle regulatory protein P27Kip1 promoter region (Luo et al., 2017). Based on the previous research background, we explored the interaction of lncRNA NR-104098 with E2F1 and their role in AML deeply. Our data showed that lncRNA NR-104098 can directly interact with E2F1. ChIP analysis showed that lncRNA NR-104098 promoted the binding of E2F1 to the EZH2 promoter region. Therefore, we conclude that the upregulation of lncRNA NR-104098 by recruiting E2F1 to the EZH2 promoter suppresses the transcription of EZH2. Our research provides a deeper understanding of lncRNA NR-104098’s transcriptional regulation of downstream genes in AML. Many types of EZH2 inhibitors have been developed on the market that have not yet been applied in clinical practice in AML patients (Martinez-Fernandez et al., 2015; Kim and Roberts, 2016). Compared with ATRA, ATPR not only has better solubility and stability but also has a better effect on inhibiting proliferation and differentiation induction (Wang H. et al., 2013; Wang N. et al., 2013; Fan et al., 2014; Hu et al., 2014; Wang et al., 2014). Our research also found that ATPR could induce G0/G1 cycle arrest in liver cancer cells and gastric cancer cells (Liu et al., 2016; Xia et al., 2017). In addition, autophagy was also effective in the differentiation of NB4 cells induced by ATPR (Li et al., 2017). ATPR induces differentiation and inhibits AML proliferation by activating ROS release and then regulating PTEN/PI3K/AKT signaling pathways (Feng et al., 2019a). ATPR promotes the release of ROS by inhibiting the formation of the EBP50/NCF1 complex and then inducing the differentiation of APL and G0/G1 phase arrest (Feng et al., 2019b). In this study, we found ATPR positively regulated lncRNA NR-104098 expression, and the induce differentiation and inhibit proliferation effect of ATPR was repressed by lncRNA NR-104098 knockdown. These results support the feasibility of using lncRNA NR-104098 to act as a promising target for development of novel anti-cancer agents in AML.

## Conclusion

**FIGURE 10:**
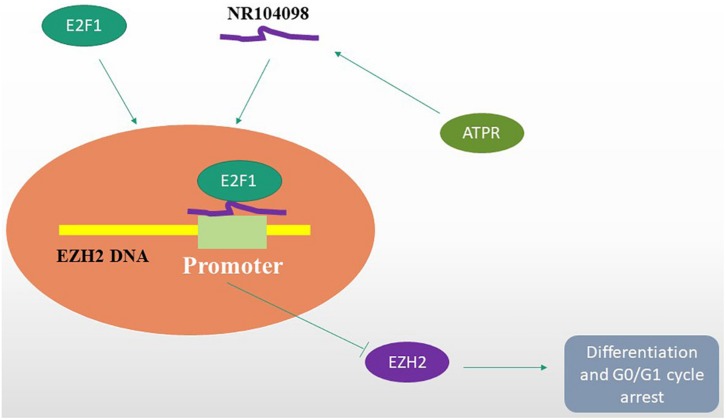
A hypothetical working model of the role of lncRNA NR-104098 in AML proliferation and differentiation. Overexpression of lncRNA NR-104098 effectively increases the binding of E2F1 to EZH2 mRNA promoter, resulting in repression of EZH2 transcription that inhibits AML cell proliferation and induces differentiation. Meanwhile, an ATRA derivative ATPR could inhibit AML cell proliferation and induce differentiation though regulating the expression of lncRNA NR-104098. The arrows refers to the role of promotion, and the symbol of “T” refers to the role of inhibition.

Overall, for the first time, this study found that lncRNA NR-104098 may be an important regulator of AML. The results of subsequent mechanistic studies showed lncRNA NR-104098 on the transcriptional level of EZH2 by enhancing the binding of E2F1 to the EZH2 promoter (Figure 10). The interaction between lncRNA NR-104098 and EZH2 plays a vital role in the proliferation and differentiation of AML. Therefore, we showed that lncRNA NR-104098 may become an attractive tumor suppressor molecule for AML treatment and extended the research on molecular mechanisms.

## Data Availability Statement

The microarray data has been deposited to the GEO – GSE145168. Other raw data supporting the conclusions of this article will be made available by the authors, without undue reservation, to any qualified researcher.

## Ethics Statement

This study was approved by the Ethics and Research Committees of Anhui Medical University and conducted in accordance with the National Institutes of Health Guide for the Care and Use of Laboratory Animals.

## Author Contributions

YF and LL conducted the project design. XX and MZ carried out the experiment. JL and SH participated in the bioinformatics analysis. TD, SZ, and YD performed the *in vivo* research part. XP and FC optimized the manuscripts. All authors participated in this study, and read and approved the final manuscript.

## Conflict of Interest

The authors declare that the research was conducted in the absence of any commercial or financial relationships that could be construed as a potential conflict of interest.

## Footnotes

1 http://www.csbio.sjtu.edu.cn/bioinf/lncLocator/index.html
