# Supplementary material for: LncRNA NR-104098 Inhibits AML Proliferation and Induces Differentiation Through Repressing EZH2 Transcription by Interacting With E2F1
Source: Front Cell Dev Biol. 2020 Mar 26;8:142. doi: 10.3389/fcell.2020.00142 (PMC7136616; doi:10.3389/fcell.2020.00142)
Supplement: Supplementary file 1 [file Table_1.DOCX]

Highlights

1. ATPR may be developed into a highly effective and low-toxic clinical first-line drug for the treatment of AML.

2. lncRNA NR-104098 was down-regulated in AML cells.

3. lncRNA NR-104098 may become a direct target for ATPR treatment of AML.

4. lncRNA NR-104098 may effectively inhibit EZH2 transcription by directly binding E2F1 and recruiting E2F1 to EZH2 promoter.

5. lncRNA NR-104098 plays an important role in ATPR-induced AML differentiation and G0/G1 phase arrest in vitro and in vivo.
